# The efficacy of add on cardiac resynchronization therapy (CRT) to symptomatic patients with heart failure with reduced ejection fraction on sacubitril/valsartan treatment as regards left ventricle reverse remodeling and functional improvement

**DOI:** 10.1186/s43044-026-00797-6

**Published:** 2026-11-10

**Authors:** Nour El Din Mohamed Nazmy, Hayam Mohamed Eldamanhory, Haitham Abdelfatah Badran, Hassan shehata hassan

**Affiliations:** Department of cardiology, Faculty of medicine, Ain Shams University, Cairo, Egypt

**Keywords:** Cardiac resynchronization therapy, Heart failure with reduced ejection fraction, Sacubitril/valsartan, Reverse remodeling

## Abstract

**Background:**

Heart failure (HF) represents a substantial worldwide healthcare burden, with nearly 26 million people affected. CRT is widely recommended for HFrEF patients with QRS duration > 150 msec, but its role in those with QRS duration of 130–149 msec remains debated. The objective was to evaluate whether adding CRT improves LV reverse remodeling and functional status in symptomatic HFrEF patients receiving maximally tolerated sacubitril/valsartan with QRS duration of 130–149 msec.

**Methods:**

This study included 100 patients with HFrEF (≤ 35%) treated with maximally tolerated sacubitril/valsartan and having LBBB morphology with QRS duration of 130–149 msec. Patients were classified into two groups: those receiving CRT and those maintained on medical therapy without CRT. Clinical status, QRS duration, and echocardiographic findings were reassessed after 6 months and compared between groups.

**Results:**

At 6 months, NYHA class improvement occurred more frequently with CRT than without CRT (37 vs. 20 patients, *P* = 0.004). The decline in end-systolic volume was greater in the CRT group (mean change, 11.42 ± 7.30 vs. 3.54 ± 6.41, *P* < 0.001). Ejection fraction also showed greater improvement with CRT (mean change, 4.24 ± 3.17 vs. 1.30 ± 2.35, *P* < 0.001). Hospitalization occurred less frequently in the CRT group (46% vs. 70%, *P* = 0.03). No deaths were observed in either group throughout follow-up.

**Conclusions:**

In symptomatic HFrEF patients receiving maximally tolerated sacubitril/valsartan with QRS duration of 130–149 msec, adjunctive CRT was associated with greater LV reverse remodeling, improved functional status, and fewer hospitalizations.

## Background

Heart failure (HF) affects nearly 26 million people worldwide and continues to place a substantial burden on global healthcare resources [1].

In older adults in the United States, approximately half of patients diagnosed with HF die within 5 years [2]. HF-related direct medical costs were estimated at $30.7 billion in 2012 and are projected to reach $69.7 billion by 2030, representing an increase of nearly 127% (2). The condition is also a frequent cause of hospital admission and is associated with exceptionally high rates of rehospitalization [3]. Reducing avoidable admissions has therefore become a major healthcare priority.

Management of chronic HF has evolved considerably over the past two decades, with important advances in both drug and device therapies. Several treatment options for HFrEF have demonstrated favorable effects on morbidity and survival. Sacubitril/valsartan is an angiotensin receptor/neprilysin inhibitor that has become an important component of HFrEF therapy. In PARADIGM-HF, a large multicenter randomized double-blind trial, treatment with sacubitril/valsartan was associated with fewer cardiovascular deaths and first admissions for worsening HF compared with enalapril [4].

Cardiac resynchronization therapy (CRT) provides an additional therapeutic option by improving electrical and mechanical synchrony and reducing inter-/intraventricular and atrioventricular dyssynchrony. Among patients with impaired LV systolic function and a prolonged QRS complex, CRT has been associated with better survival and fewer HF-related admissions [5]. These favorable outcomes are accompanied by reverse LV remodeling and improvement in LVEF [6].

According to European Society of Cardiology guidelines, CRT has a Class I indication in patients with HFrEF and QRS duration > 150 msec. The benefit is less clearly established when QRS duration is 130–149 msec, where available evidence remains inconsistent and the recommendation is Class II [7]. Accordingly, this study assessed the effect of adding CRT to maximally tolerated sacubitril/valsartan on LV reverse remodeling and functional improvement in symptomatic HFrEF patients with QRS duration of 130–149 msec.

## Patients and methods

### Study design and setting

A prospective observational design was adopted for this study, which was performed at Ain Shams University Hospital, Cairo, Egypt, from October 2022 through November 2024. The study population comprised 100 patients with HFrEF who were categorized into two study groups and followed for 6 months.

### Eligibility criteria

Enrollment required the presence of dilated or ischemic cardiomyopathy, LVEF ≤ 35%, NYHA functional class III–IV, sinus rhythm, and LBBB morphology with QRS duration between 130 and 149 msec. Participants were also required to be receiving optimal medical treatment with maximally tolerated sacubitril/valsartan, beta-blockers, and MRAs. Patients were not eligible if they were ≥ 75 years old, had eGFR < 30 mL/min/1.73 m², serum potassium > 5.2 mmol/L at screening, previous angioedema or intolerance to ACE inhibitors or ARBs, surgically correctable valvular disease, anticipated coronary revascularization, or blood pressure that precluded the use of sacubitril/valsartan.

### Study groups

#### Patients were categorized into two groups

CRT group: 50 patients receiving maximally tolerated sacubitril/valsartan in addition to CRT.

No-CRT group: 50 patients treated with maximally tolerated sacubitril/valsartan without CRT.

All participants underwent clinical assessment, standard ECG, and echocardiographic examination at baseline and again after 6 months.

Standard echocardiography was performed according to American Society of Echocardiography recommendations, using 2D, M-mode, and Doppler imaging. Examinations were obtained with a GE Vivid S5N Ver. 10.3.0 b.114 ultrasound system equipped with an RS3 probe.

All images were acquired by a senior operator and subsequently interpreted by experienced independent cardiologists who were blinded to group allocation.

#### CRT implantation and device programming

- Transvenous lead insertion was performed through the subclavian vein with fluoroscopic guidance.
- The RV lead was placed at the RV apex.
- The LV lead was introduced through the CS and deployed in a lateral or posterolateral venous branch.
- Programming assessment included VV delay, RV pacing threshold, RA and RV lead impedance, CS pacing threshold and impedance, RA and RV sensing amplitudes, paced AV delay, and output settings for the RA, RV, and CS leads at 0, 3- and 6-months post implantation.

A standardized definition of CRT response remains lacking despite long-standing clinical use. Criteria applied in randomized trials are not identical to those commonly used in routine practice. Trials generally evaluate predefined clinical events, whereas everyday assessment often relies on less uniform measures of improvement.

Response to CRT may also vary according to patient expectations. Individuals with severe HF initially seek symptomatic relief and better quality of life. Once these goals are achieved, attention may shift toward reducing hospital admissions, improving social activity, and extending survival.

In the present study, response was classified as follows

- Clinical response: improvement by ≥ 1 NYHA functional class.
- Echocardiographic response: increase in LVEF by ≥ 5% accompanied by ≥ 15% reduction in LVES diameter.

### Statistical methods

IBM SPSS version 23 was used for data management and statistical evaluation. Continuous variables were expressed as mean ± SD or median (IQR), depending on distribution, while categorical variables were summarized as frequencies and percentages. Chi-square and Fisher’s exact tests were used for categorical comparisons. Paired and independent t-tests were applied to continuous variables involving two groups, whereas One-Way ANOVA was used when comparing more than two groups. Correlations between quantitative variables were assessed using Spearman’s test. A 95% confidence interval was used, with *P* > 0.05 regarded as non-significant, *P* < 0.05 as significant, and *P* < 0.01 as highly significant.

## Results

One hundred symptomatic patients diagnosed with HFrEF on maximum tolerated doses of Sacubitril/Valsartan therapy were enrolled with 50 patients in each group.

There was no significant difference between CRT group and no-CRT group in baseline demographic data, risk factors, etiology of cardiomyopathy and QRS complex duration except for hypertension which was significantly higher in no-CRT group (no = 39 patients (pts). versus 29 pts in CRT group, *P* = 0.032). Table 1.

**Table 1:** Comparison between CRT group and no-CRT group regarding demographic data, risk factors, etiology of cardiomyopathy and QRS duration

		CRT group	No-CRT group	P-value	Significance
		Number = 50	Number = 50		
Age	Mean ± SD	63.32 ± 5.63	61.20 ± 6.90	0.119	NS
	Range	52–74	46–74		
Gender	Male	32 (64.0%)	27 (54.0%)	0.309	NS
	Female	18 (36.0%)	23 (46.0%)		
Body mass index	Mean ± SD	30.50 ± 3.86	30.10 ± 3.93	0.573	NS
	Range	24–43	24–43		
Diabetes	No	21 (42.0%)	19 (38.0%)	0.683	NS
	Yes	29 (58.0%)	31 (62.0%)		
Hypertension	No	21 (42.0%)	11 (22.0%)	0.032	S
	Yes	29 (58.0%)	39 (78.0%)		
Cardiomyopathy	DCM	24 (48.0%)	20 (40.0%)	0.420	NS
	ICM	26 (52.0%)	30 (60.0%)		
QRS duration (msec)	Mean ± SD	134.58 ± 5.37	134.18 ± 5.32	0.695	NS
	Range	130–149	130–149		

DCM= dilated cardiomyopathy, ICM: ischemic cardiomyopathy, NS = non-significant, S= significant, SD= standard deviation

A Multivariable logistic regression analysis was performed, and hypertension was not independently associated with response (adjusted OR 0.70, 95% CI 0.24–2.08; *p* = 0.525).

Both groups were on the same baseline medical treatment including sacubitril/valsartan, Beta blockers, mineralocorticoid receptor antagonists (MRAs). No patients were on Sodium Glucose Transport protein 2 inhibitors (SGLT2 inhibitors).

**Fig. 1:**
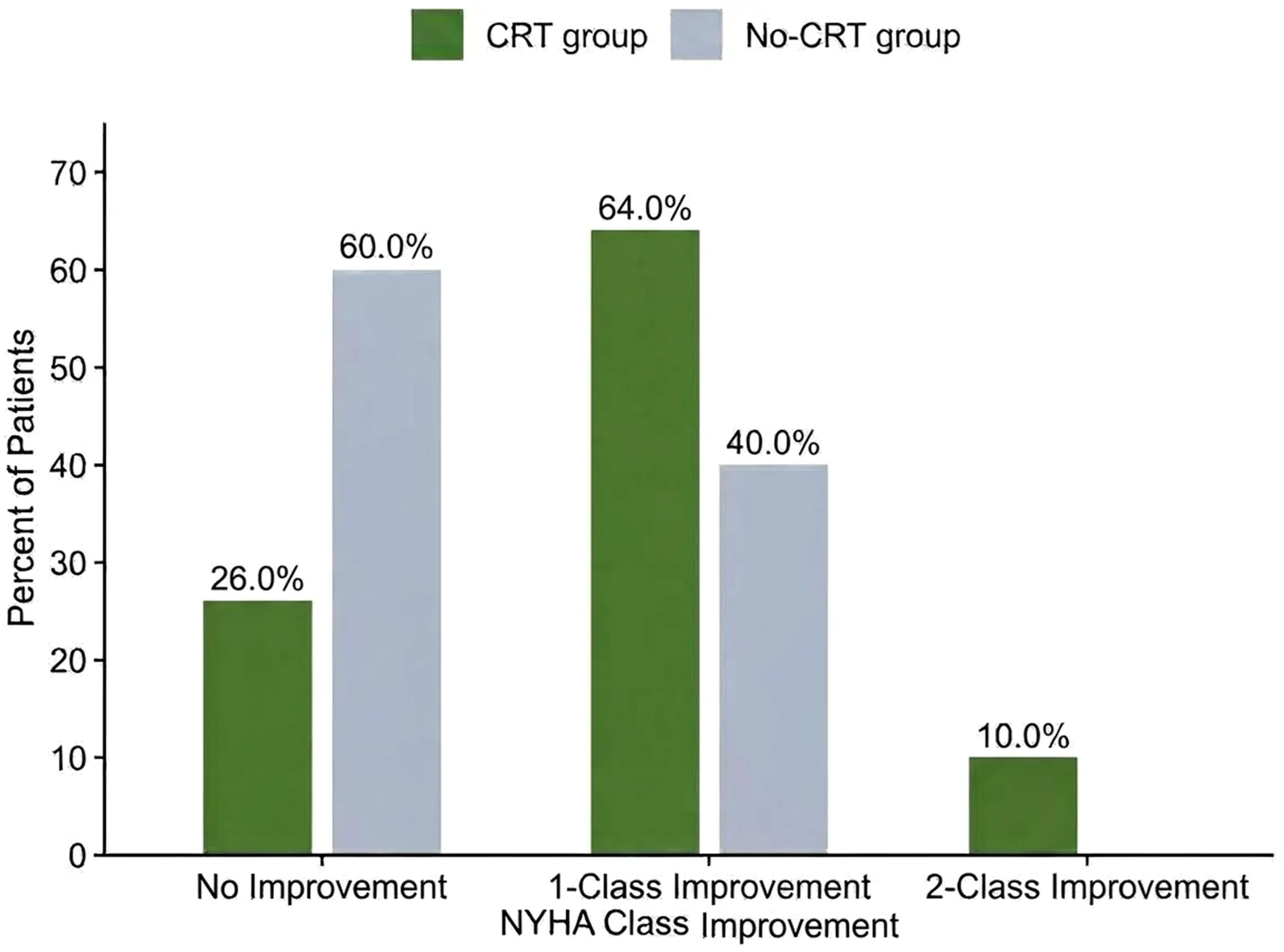
Comparison between CRT group and no-CRT group regarding NYHA class improvement CRT= cardiac synchronization therapy, NYHA = New York Heart Association

During follow-up, the CRT group demonstrated a highly significant improvement in functional capacity according to NYHA classification compared with the no-CRT group (*P* = 0.004; Table 2). Improvement by one NYHA class was observed in 32 patients (64%) in the CRT group versus 20 patients (40%) in the no-CRT group, while improvement by two classes occurred in 5 patients (10%) in the CRT group and none in the no-CRT group. Figure 1.

**Table 2:** Comparison between CRT group and no-CRT group regarding functional improvement (NYHA classification)

NYHA improvement	CRT group	No-CRT group	P-value	Significance
	Number = 50	Number = 50		
No	13 (26.0%)	30 (60.0%)	0.004	HS
One class	32 (64.0%)	20 (40.0%)		
Two class	5 (10.0%)	0 (0.0%)		

HS= highly significant, NYHA = New York Heart Association

The functional improvement of the patients in CRT group was associated with improvement in the echocardiographic data illustrated by End-systolic volume (ESV) reduction and ejection fraction (EF) improvement. Table 3.

**Table 3:** Comparison between CRT group and no-CRT group regarding echocardiographic improvement measured by ESV reduction and EF

		CRT group	No-CRT group	P-value	Significance
		Number = 50	Number = 50		
ESV reduction (%)	Mean ± SD	11.42 ± 7.30	3.54 ± 6.41	< 0.001	HS
	Range	0–20	0–16		
EF improvement by Simpsons method (%)	Mean ± SD	4.24 ± 3.17	1.30 ± 2.35	< 0.001	HS
	Range	0–12	0–6		

EF= ejection fraction, ESV = end systolic volume, HS= highly significant, SD= standard deviation

**Fig. 2:**
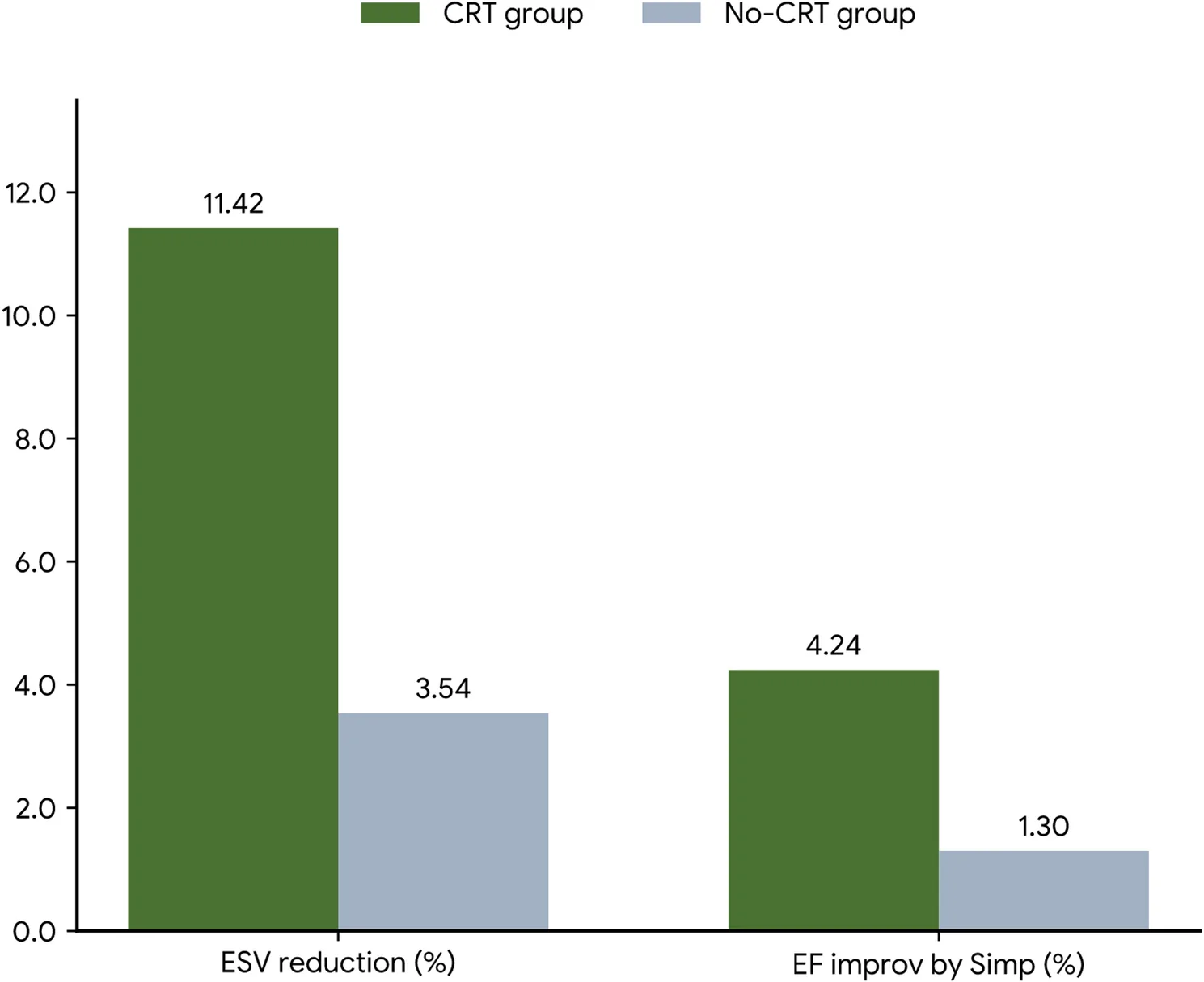
Comparison between CRT group and no-CRT group regarding ESV reduction (%) and EF improvement by Simpsons (%) CRT= cardiac synchronization therapy, EF= ejection fraction, ESV = end systolic volume

ESV was significantly reduced in the CRT group compared with the no-CRT group (mean change: 11.42 ± 7.30 vs. 3.54 ± 6.41, *P* < 0.001). Similarly, LVEF improved significantly in the CRT group (mean change: 4.24 ± 3.17% vs. 1.30 ± 2.35%, *P* < 0.001). Figure 2.

**Fig. 3:**
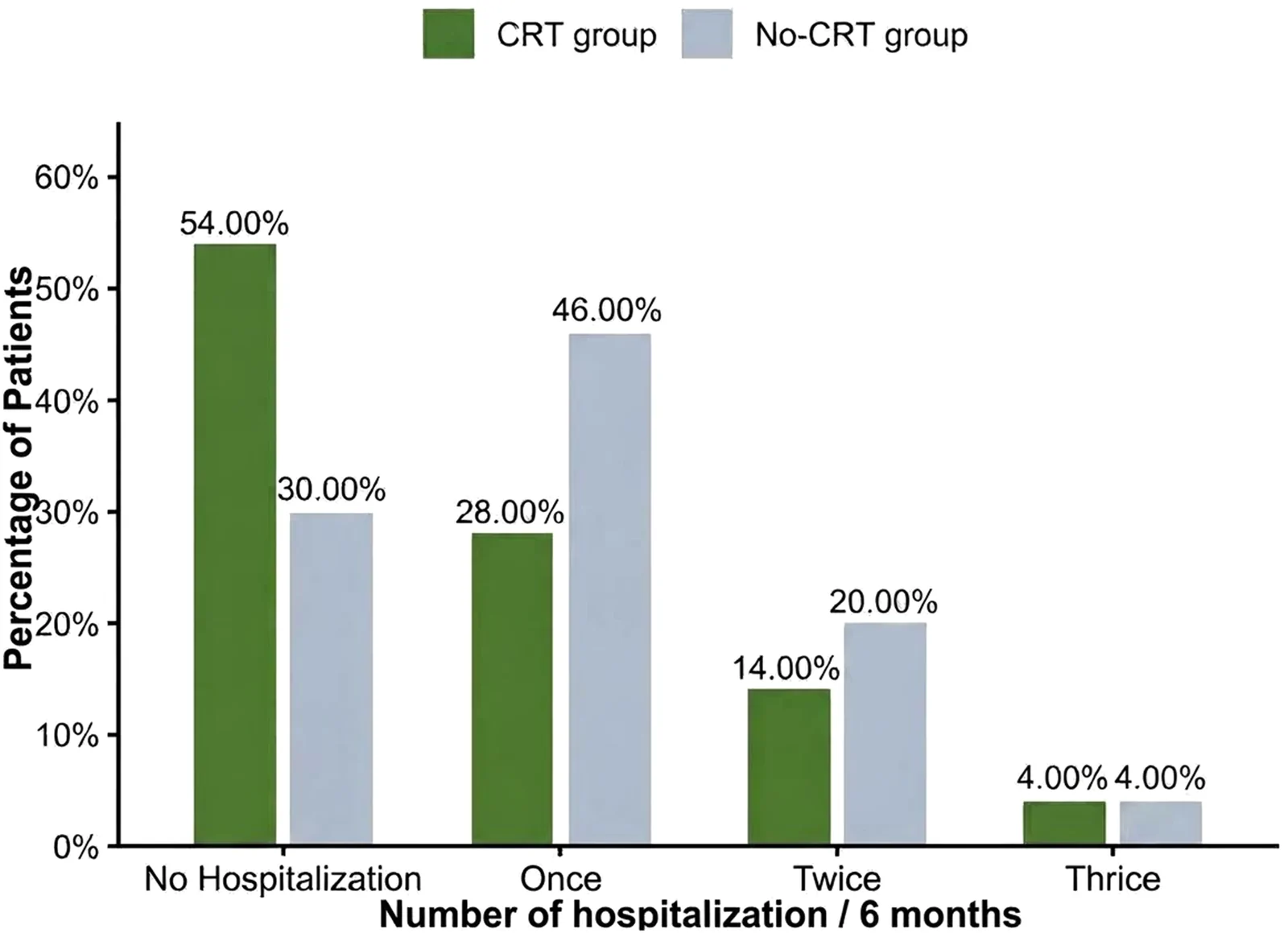
Comparison between CRT group and no-CRT group regarding 6 month-hospitalization CRT= cardiac synchronization therapy

As regards the 6-month hospitalization frequency, less hospitalization frequency was observed in CRT group in comparison to no-CRT group and no mortality was recorded in both groups during the follow up period. Table 4; Fig. 3.

**Table 4:** Comparison between CRT group and no-CRT group regarding hospitalization and mortality

		CRT group	No-CRT group	*P*-value	Significance
		Number = 50	Number = 50		
6- month hospitalization	None	27 (54.0%)	15 (30.0%)	0.03	S
	Once	14 (28.0%)	23 (46.0%)		
	Twice	7 (14.0%)	10 (20.0%)		
	Three	2 (4.0%)	2 (4.0%)		
Mortality	No	50 (100.0%)	50 (100.0%)	–	–

S= significant

In CRT group, 34 patients (68%) were responders to add on CRT with 16 patients (32%) were non-responders.

**Fig. 4:**
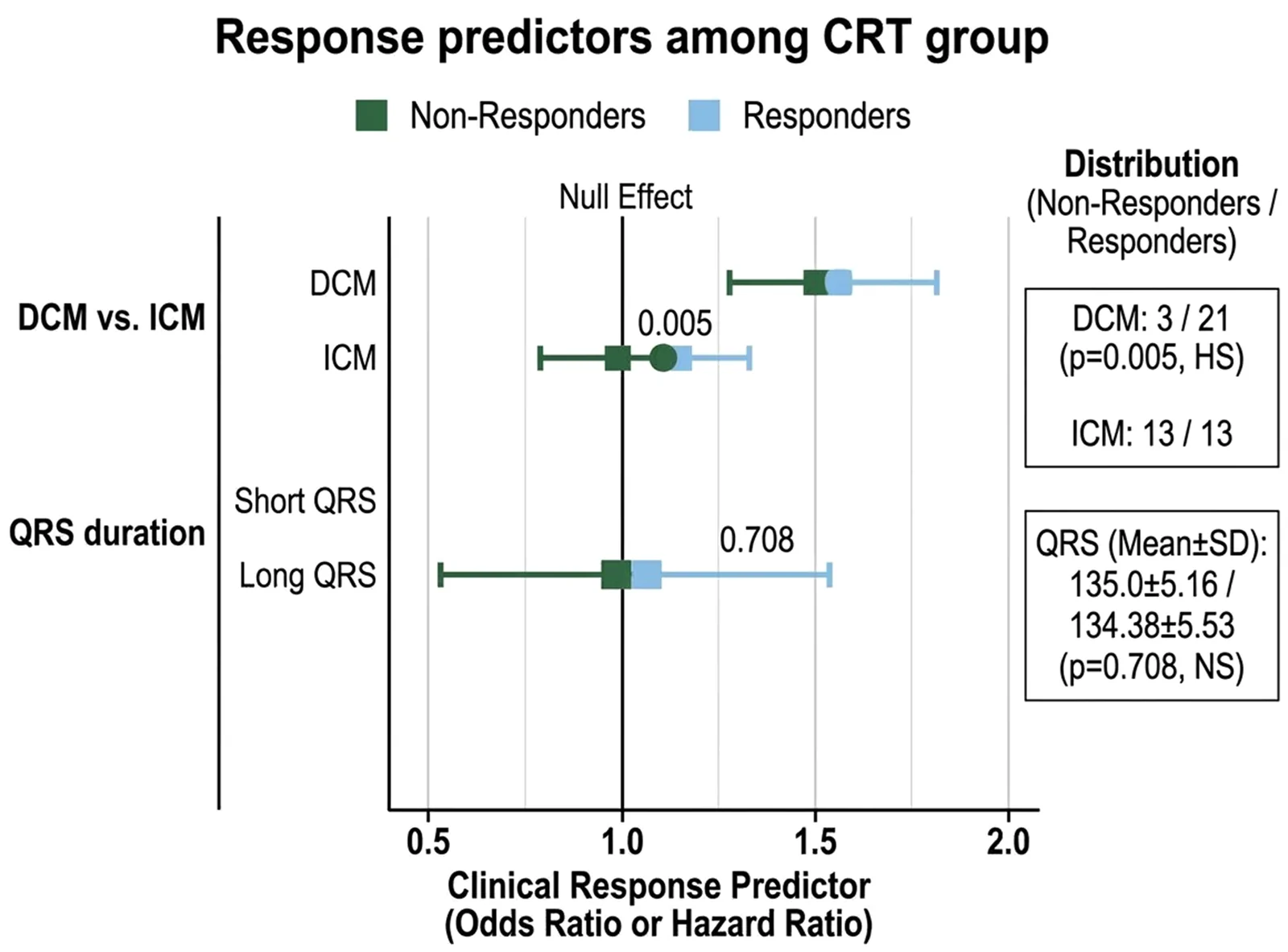
Response predictors among CRT group

No significant difference was denoted between the responders and the non-responders among CRT group as regards the baseline demographic data, risk factors, baseline QRS duration and medical treatment **(**Table 5**)**. Most of the responders were of DCM etiology (21 pts., 61.8%). However, most of the non-responders were of ICM etiology (13 pts., 81.2%). Figure 4.

**Table 5:** Comparison between CRT responders and non-responders

		Non-responders	Responders		
		Number = 16	Number = 34		
Age	Mean ± SD	62.75 ± 5.05	63.59 ± 5.93	0.628	NS
	Range	53–69	52–74		
Gender	Male	12 (75.0%)	20 (58.8%)	0.266	NS
	Female	4 (25.0%)	14 (41.2%)		
Body mass index	Mean ± SD	31.94 ± 4.82	29.82 ± 3.17	0.070	NS
	Range	26–43	24–37		
Diabetes	No	6 (37.5%)	15 (44.1%)	0.658	NS
	Yes	10 (62.5%)	19 (55.9%)		
Hypertension	No	7 (43.8%)	14 (41.2%)	0.863	NS
	Yes	9 (56.2%)	20 (58.8%)		
Cardiomyopathy	DCM	3 (18.8%)	21 (61.8%)	0.005	HS
	ICM	13 (81.2%)	13 (38.2%)		
Diuretics	No	0 (0.0%)	2 (5.9%)	0.322	NS
	Yes	16 (100.0%)	32 (94.1%)		
QRS duration	Mean ± SD	135.0 ± 5.16	134.38 ± 5.53	0.708	NS
	Range	130–140	130–149		

CRT= cardiac synchronization therapy, DCM= dilated cardiomyopathy, HS=Highly significant, ICM: ischemic cardiomyopathy, NS = non-significant, SD= standard deviation

Regarding functional improvement measured by NYHA classification; 29 patients (85.3%) improved by one class among responders vs. only 3 patients (18.7%) among non-responders. And 5 patients (14.7%) improved by 2 classes among the responders. Table 6.

**Table 6:** Comparison between CRT responders and non-responders regarding functional improvement, echocardiographic improvement and 6-months hospitalization

		Non-responders	Responders	P-value	Significance
		Number = 16	Number = 34		
NYHA improvement	No	13 (81.2%)	0 (0.0%)	< 0.001	HS
	One class	3 (18.7%)	29 (85.3%)		
	Two class	0 (0.0%)	5 (14.7%)		
6-month hospitalization	None	1 (6.2%)	26 (76.5%)	< 0.001	HS
	Once	6 (37.5%)	8 (23.5%)		
	Twice	7 (43.8%)	0 (0.0%)		
	Three	2 (12.5%)	0 (0.0%)		

HS= highly significant, IQR= interquartile range, NYHA= New York Heart Association

A significant difference was observed as regards the frequency of hospitalization during the 6 months follow up period. Less hospitalization frequency was denoted in the responders vs. non-responders (None: 26 pts. vs. 1 pt. - Once: 8 pts. vs. 6 pts. - twice: 0 pts. vs. 7 pts. - Thrice: 0 pts. vs. 2 pts., *P* < 0.001). Table 6.

## Discussion

CRT has become an important therapeutic option in HF management. It acts by improving ventricular synchrony, which is impaired in up to one-third of patients with severe symptomatic systolic HF [8]. More coordinated myocardial contraction enhances mechanical performance and may translate into better clinical status and improved survival.

### Impact of QRS morphology on response to medical therapy

Among patients managed without CRT, despite maximally tolerated pharmacological therapy, improvement in EF and ESV over follow-up was limited, with mean changes of 1.30 ± 2.35 and 3.54 ± 6.41, respectively. Importantly, treatment in this group included sacubitril/valsartan rather than ACE inhibitors or ARBs.

Edward et al. [9] showed in 2018 that LBBB was associated with the least recovery in LVEF during optimal medical treatment when compared with WQRS and NQRS. LVEF exceeded 35% in 23% of patients with LBBB, compared with 27% in WQRS and 43% in NQRS. Corresponding mean LVEF changes were 3.41 ± 9.43, 4.86 ± 9.22, and 9.50 ± 12.17, respectively. Furthermore, pivotal trials of sacubitril/valsartan, ivabradine, and SGLT2 inhibitors included relatively few patients meeting CRT criteria, limiting the evidence supporting prolonged medical optimization before implantation. This may favor earlier CRT consideration in LBBB, where pharmacological recovery of LVEF appears less pronounced.

Villaschi et al. [10] assessed the relationship between CRT timing after SMT and subsequent outcomes. Implantation within < 3 months of SMT was associated with a 9% reduction in cardiovascular death compared with implantation at 3–9 months (*P* = 0.045). In contrast, CRT performed after > 9 months was associated with a 13% increase in cardiovascular death and HF hospitalization compared with the 3–9-month interval (*P* = 0.040). These data emphasize the potential prognostic value of avoiding unnecessary delay in CRT implantation.

QRS duration should also be considered alongside treatment timing when selecting patients for CRT. Deferring implantation until LBBB reaches a QRS duration ≥ 150 msec may postpone a potentially beneficial intervention. Patients with QRS duration of 130–149 msec may still gain clinical benefit and therefore merit consideration for earlier CRT.

### Influence of QRS morphology and duration on response to CRT

In patients with LBBB and a QRS interval of 130–149 msec, CRT was associated with meaningful symptomatic and echocardiographic improvement. At follow-up, ≥ 1-class improvement in NYHA status was observed in 64% of the CRT group compared with 40% of the no-CRT group, whereas a 2-class improvement was documented in 10% versus 0%, respectively. Patients treated with CRT also had a larger gain in LVEF (4.24 ± 3.17 vs. 1.30 ± 2.35), a greater fall in LV end-systolic volume (11.42 ± 7.30 vs. 3.54 ± 6.41), and fewer hospital admissions.

The MADIT-CRT trial [11] demonstrated a comparable pattern. In participants with LBBB, CRT lowered the risk of HF or death by 53% relative to ICD alone (hazard ratio 0.47; *P* < 0.001). A greater increase in LVEF was also observed with CRT (11.9 ± 5.1% vs. 3.4 ± 3.0%). Together, these data indicate that the presence of LBBB may identify electrical dyssynchrony more effectively than QRS width alone. Therefore, CRT may remain beneficial in symptomatic systolic dysfunction with LBBB even when QRS duration has not reached 150 msec.

The RAFT trial by Birnie et al. [12] showed that, among patients with LBBB, the advantage of ICD-CRT became greater as QRS duration increased. Nevertheless, benefit extended across the full QRS range without an obvious threshold below which CRT should be withheld. A possible delay in benefit among patients with QRS ≥ 160 msec was noted, although this observation requires further evaluation.

Our findings differ from the meta-analysis by Sipahi et al. [13], which reported a reduction in clinical events with CRT only when QRS duration exceeded 150 msec (risk ratio 0.60; 95% CI, 0.53–0.67; *P* < 0.001), whereas no significant benefit was demonstrated with QRS duration of 120–150 msec (relative risk 0.95; 95% CI, 0.82–1.20; *P* = 0.49), irrespective of NYHA class or ICD use. However, that analysis was based on aggregate rather than patient-level data and therefore could not adequately examine the combined influence of QRS morphology and duration. In our cohort, all patients had LBBB with intermediate QRS prolongation, which may partly explain the discrepancy.

Our study primarily evaluated LV reverse remodeling and showed significant favorable changes, with an increase in LVEF (mean change 4.24 ± 3.17; *P* < 0.001) and a reduction in LVESV (mean change 11.42 ± 7.30; *P* < 0.001).

These results are consistent with the REVERSE trial [14], which enrolled patients with NYHA class I–II HF, QRS ≥ 120 msec, and LVEF ≤ 40%. Adding CRT to optimal medical therapy reduced HF hospitalization (hazard ratio 0.47; *P* = 0.03) and improved LV geometry and function. LV end-systolic volume index decreased markedly in the CRT-On group compared with CRT-Off (-18.4 ± 29.5 vs. -1.3 ± 23.4 ml/m²; *P* < 0.0001).

In contrast, REVERSE did not demonstrate significant gains in quality of life or exercise capacity. This discrepancy may reflect its inclusion of patients with milder baseline symptoms (NYHA I–II) and some with QRS duration of 120–130 msec, whereas all patients in our study had QRS > 130 msec.

### Evaluating response to cardiac resynchronization therapy

In 2020, a joint position statement was published with a call for change to the traditional concept of “response” to CRT, often defined by arbitrary cut-offs such as ≥ 15% LV end-systolic volume reduction and symptomatic improvement, which led to overemphasis on non-response [15]. As patients without significant LV reverse remodeling can still derive symptomatic and hemodynamic benefits. Labelling patients as “responders” can also risk suboptimal follow-up care. Given that HF is incurable, CRT should not be viewed as a binary “response” therapy but rather as a disease-modifying intervention aimed at slowing progression and reducing morbidity and mortality.

In our study, 68% of the patients with implanted CRT were echocardiographic responders. 74% of the patients were clinical responders. Among 16 non-responders, 3 patients (18.7%) showed clinical improvement without showing echocardiographic criteria of LV reverse remodeling.

Our results align with the PROSPECT trial [16], which evaluated 498 patients with standard CRT indications. Positive response, defined as improvement in the clinical composite score or ≥ 15% reduction in LV end-systolic volume at 6 months, was observed in 69% and 56% of patients, respectively, confirming the clinical and echocardiographic benefits of CRT.

Our study demonstrates a highly significant relationship between underlying etiology and CRT response status (*P* = 0.005), with 81.2% of non-responders exhibiting an ischemic etiology (ICM) compared to a predominantly non-ischemic dilated substrate (61.8% DCM) among responders. This divergence highlights a critical pathophysiological barrier. While the diffuse interstitial substrate of DCM preserves enough contractile reserve to allow structural reverse remodelling, the dense, localized transmural scar tissue characteristic of ICM acts as an inexcitable electrical sink. In this intermediate QRS cohort—where baseline dyssynchrony is inherently marginal—scar-mediated conduction blocks and tissue non-viability effectively neutralize the therapeutic benefit of biventricular pacing, disproportionately driving non-response. This was coherent with landmark clinical trial data, such as the MADIT-CRT subset analyses [17].

This study has several limitations that should be considered when interpreting its findings. First, it was a single-center observational study, which may limit the generalizability of the results to other settings and populations. In addition, the relatively limited sample size may have reduced the statistical power to detect smaller differences between groups. In terms of the absence of SGLT2i therapy among the study population, our study was constrained by socioeconomic and logistical limitations that precluded the implementation of a strict, protocol-mandated regimen. Also, the follow-up period was restricted to 6 months, preventing assessment of the longer-term effects and durability of the observed outcomes. Furthermore, sacubitril/valsartan dosing was not uniform across participants, which may have introduced variability in treatment response. ECG and echocardiographic measurements may also have been subject to interobserver variability. Finally, the timing of post-CRT assessment was not identical for all patients, which may have influenced the observed degree of improvement and introduced variability in the assessment of treatment response.

## Conclusions

Our data suggest that CRT is associated with favourable left ventricular reverse remodelling, improved functional status, and a reduction in hospitalizations among symptomatic HFrEF patients with an intermediate QRS duration of 130–149 msec.

## Data Availability

The datasets used and/or analyzed during the current study are available from the corresponding author on reasonable request.

## Abbreviations

ACE: Angiotensin converting enzyme
ARBs: Angiotensin receptor blockers
AV: Atrioventricular
BB: Beta blocker
CRT: Cardiac resynchronization therapy
CS: Coronary sinus
DCM: Dilated cardiomyopathy
EF: Ejection fraction
ESV: End systolic volume
HF: Heart failure
HfrEF: Heart failure with reduced ejection fraction
ICD: Implantable cardioverter defibrillator
ICM: Ischemic cardiomyopathy
LBBB: Left bundle branch block
LV: Left ventricle
LVEF: Left ventricular ejection fraction
LVES: Left ventricle end-systolic
MRAs: Mineralocorticoid receptor antagonists
NYHA: New York Heart Association
Pts: Patients
RA: Right atrium
RV: Right ventricle
SMT: Stable medical treatment

## References

[1] Savarese G, Lund LH (2017) Global Public Health Burden of Heart Failure. Card Fail Rev Apr 3(1):7–11. 10.15420/cfr.2016:25:2 PMC5494150 28785469

[2] Heidenreich PA, Albert NM, Allen LA, Bluemke DA, Butler J, Fonarow GC et al (2013) Forecasting the impact of heart failure in the United States: a policy statement from the American Heart Association. Circ Heart Fail May; 6(3):606–619. 10.1161/HHF.0b013e318291329a PMC3908895 23616602

[3] Jencks SF, Williams MV, Coleman EA (2009) Rehospitalizations among patients in the Medicare fee-for-service program. N Engl J Med. Apr 2;360(14):1418-28. 10.1056/NEJMsa0803563 19339721

[4] McMurray JJ, Packer M, Desai AS, Gong J, Lefkowitz MP, Rizkala AR et al (2014) Angiotensin-neprilysin inhibition versus enalapril in heart failure. N Engl J Med. Sep 11;371(11):993–1004. 10.1056/NEJMoa1409077 25176015

[5] McMurray JJ, Adamopoulos S, Anker SD, Auricchio A, Böhm M, Dickstein K et al (2012) ESC Guidelines for the diagnosis and treatment of acute and chronic heart failure 2012. Eur Heart J Jul 33(14):1787–1847. 10.1093/eurheartj/ehs104 22611136

[6] Ponikowski P, Voors AA, Anker SD, Bueno H, Cleland JG, Coats AJ et al (2016) 2016 ESC Guidelines for the diagnosis and treatment of acute and chronic heart failure. Eur Heart J. Jul 14;37(27):2129 – 200. 10.1093/eurheartj/ehw128 27894487

[7] McDonagh TA, Metra M, Adamo M, Gardner RS, Baumbach A, Böhm M et al (2021) 2021 ESC Guidelines for the diagnosis and treatment of acute and chronic heart failure. Eur Heart J. Sep 21;42(36):3599 – 726. 10.1093/eurheartj/ehab368 34447992

[8] McAlister FA, Ezekowitz J, Hooton N, Vandermeer B, Spooner C, Dryden DM et al (2007) Cardiac resynchronization therapy for patients with left ventricular systolic dysfunction: a systematic review. JAMA. Jun 13;297(22):2502-14. 10.1001/jama.297.22.2502 17565085

[9] Sze E, Samad Z, Dunning A, Campbell KB, Loring Z, Atwater BD et al (2018) Impaired recovery of left ventricular function in patients with cardiomyopathy and left bundle branch block. J Am Coll Cardiol. Jan 23;71(3):306 – 17. 10.1016/j.jacc.2017.11.020 PMC8352572 29348023

[10] Villaschi A, Basile C, Benson L, Gatti P, Abraham W, Böhm M et al (2025) Timing of Cardiac Resynchronization Therapy Following Stable Medical Therapy in Patients With Heart Failure. JACC Heart Fail Oct 13(10):102515. 10.1016/j.jchf.2025.102515 40542793

[11] Zareba W, Klein H, Cygankiewicz I, Hall WJ, McNitt S, Brown M et al (2011) Effectiveness of cardiac resynchronization therapy by QRS morphology in the multicenter automatic defibrillator implantation trial–cardiac resynchronization therapy (MADIT-CRT). Circulation. Mar 15;123(10):1061-72. 10.1161/CIRCULATIONAHA.110.960898 21357819

[12] Birnie DH, Ha A, Higginson L, Sidhu K, Green M, Philippon F et al (2013) Impact of QRS morphology and duration on outcomes after cardiac resynchronization therapy: Results from the Resynchronization-Defibrillation for Ambulatory Heart Failure Trial (RAFT). Circ Heart Fail Nov 6(6):1190–1198. 10.1161/CIRCHEARTFAILURE.113.000380 23995437

[13] Sipahi I, Carrigan TP, Rowland DY, Stambler BS, Fang JC. Impact of QRS duration on clinical event reduction with cardiac resynchronization therapy: meta-analysis of randomized controlled trials. Arch Intern Med. Sep 12;171(16):1454-62. 10.1001/archinternmed.2011.247. 21670335

[14] Linde C, Abraham WT, Gold MR, St John Sutton M, Ghio S, Daubert C et al (2011) (2008) Randomized trial of cardiac resynchronization in mildly symptomatic heart failure patients and in asymptomatic patients with left ventricular dysfunction and previous heart failure symptoms. J Am Coll Cardiol. Dec 2;52(23):1834-43. 10.1016/j.jacc.2008.08.027. 19038680

[15] Mullens W, Auricchio A, Martens P, Witte K, Cowie R, Delgado V et al (2020) Optimized implementation of cardiac resynchronization therapy: a call for action for referral and optimization of care. Eur J Heart Fail Nov 22(12):2349–2369. 10.1002/ejhf.2046 33136300

[16] Chung ES, Leon AR, Tavazzi L, Sun JP, Nihoyannopoulos P, Merlino J et al (2008) Results of the predictors of response to CRT (PROSPECT) trial. Circulation. May 20;117(20):2608-16. 10.1161/CIRCULATIONAHA.107.743120 18458170

[17] Barsheshet A, Goldenberg I, Moss AJ, Eldar M, Huang DT, McNitt S et al (2011) Response to preventive cardiac resynchronization therapy in patients with ischaemic and nonischaemic cardiomyopathy in MADIT-CRT. Eur Heart J Jul 32(13):1622–1630. 10.1093/eurheartj/ehq407 21075774

